# Wiskott-Aldrich syndrome: clinical, immunological, and genetic characterization of the first Moroccan cohort

**DOI:** 10.3389/fped.2026.1908492

**Published:** 2026-08-03

**Authors:** Mounia El Alaoui El Hanafi, Ibtihal Benhsaien, Khadija Maani, Jalila El Bakkouri, Abdelhakim Youssef Benmoussa, Noufissa Benajiba, Abdessalam Outabounte, Abderrahmane Errami, Fatima Ailal, Ahmed Aziz Bousfiha, Abdelhak Abkari

**Affiliations:** 1 Department of Pediatric Hematology (P3), Abderrahim El Harouchi Mother-Child Hospital, Ibn Rochd University Hospital Center, Casablanca, Morocco; 2 Laboratory of Clinical Immunology, Infection and Autoimmunity (LICIA), Faculty of Medicine and Pharmacy, Hassan II University of Casablanca, Casablanca, Morocco; 3 Department of Clinical Immunology and Pediatric Infectious Diseases (P1), Abderrahim El Harouchi Mother-Child Hospital, Ibn Rochd University Hospital Center, Casablanca, Morocco; 4 Immunology Laboratory, University Hospital Center Ibn Rochd, Casablanca, Morocco; 5 Immunopathology, Immunomonitoring, and Immunotherapy Laboratory, Mohammed VI University of Health Sciences (UM6SS), Casablanca, Morocco

**Keywords:** eczema, genetic testing, hematopoietic stem cell transplantation, inborn errors of immunity, recurrent infections, thrombocytopenia, wiskott–Aldrich syndrome

## Abstract

**Background:**

Wiskott–Aldrich syndrome (WAS) is a rare X-linked inborn error of immunity characterized by thrombocytopenia, eczema, and recurrent infections, with additional risks of autoimmunity and malignancy. Country-level data from North Africa are scarce. We report the first genetically confirmed Moroccan series of WAS, describing diagnostic pitfalls, clinical spectrum, and molecular findings.

**Methods:**

We conducted a mixed retrospective-prospective observational cohort study of male patients with molecularly confirmed Wiskott-Aldrich syndrome managed at a Moroccan tertiary referral center between January 2017 and September 2024. Clinical features, hematologic and immunologic data, management, and outcomes were collected from hospital records and available follow-up.

**Results:**

Ten male patients were included. Thrombocytopenia was present in all patients (100%), followed by recurrent infections in 9/10 patients (90%) and eczema (90%). Autoimmune complications occurred in 2/10 patients (20%), manifesting as systemic lupus erythematosus with lupus-nephritis-compatible disease in one case and autoimmune hemolytic anemia in the other. Mean platelet volume (MPV) was available in 9/10 patients and was reduced in 2/9 (22.2%). Molecular analysis identified seven distinct pathogenic/likely pathogenic variants: two nonsense (p.Gly322* and p.Arg321*), one splice-site (c.735-2A > T), two frameshift (p.Pro330Leufs*115 and p.Arg431Serfs*64), and two missense (p.Val50Asp and p.Phe128Cys). The p.Gly322* variant recurred in three related patients, including twin brothers, while c.735-2A > T was found in two unrelated individuals, highlighting both familial recurrence and allelic heterogeneity. Truncating and splice-site variants predominated in this cohort (8/10, 80%), consistent with previously reported classic WAS cohorts. One child underwent hematopoietic stem cell transplantation (HSCT) abroad with sustained remission, whereas the others remain on supportive therapy with intravenous immunoglobulin and antimicrobial prophylaxis.

**Conclusions:**

This first genetically confirmed Moroccan case series expands the clinical and molecular spectrum of Wiskott–Aldrich syndrome in North Africa. Our findings highlight the marked clinical and genetic heterogeneity of WAS and underscore the importance of early molecular diagnosis to guide appropriate management, genetic counseling, and timely referral for curative therapy in resource-limited settings.

## Introduction

Wiskott–Aldrich syndrome (WAS) is a rare X-linked inborn error of immunity (IEI) characterized by the classical triad of microthrombocytopenia, eczema, and recurrent infections, although its clinical presentation varies widely in severity and expression (1, 2). Patients also face markedly increased risk of autoimmune complications and hematologic malignancies (3, 4). The global incidence of WAS is estimated at 1 to 10 affected males per million live births, highlighting both its rarity and the ongoing challenges in diagnosis, particularly in resource-limited regions (2, 5). Without timely recognition and curative therapy, the prognosis of classic WAS remains poor, with progressive morbidity and reduced life expectancy (2, 5).

The pathogenesis of WAS results from pathogenic variants in the *WAS* gene, which encodes the Wiskott-Aldrich syndrome protein (WASp), a cytosolic protein expressed in hematopoietic cells and essential for actin cytoskeleton remodeling and immune synapse formation (6). Defects in WASp disrupt multiple cellular pathways including T-cell activation, B-cell receptor signaling, dendritic-cell migration, and platelet morphogenesis, explaining the multisystemic clinical presentation (7). The wide phenotypic spectrum of WAS is fundamentally linked to the molecular impact of the variant: null mutations typically cause the severe classic WAS phenotype, while specific missense variants may result in the milder allelic form X-linked thrombocytopenia (XLT) (8). X-linked neutropenia (XLN) is a distinct entity caused by gain-of-function variants that disrupt autoinhibition and produce constitutively active WASp, resulting in severe congenital neutropenia with a normal platelet count (9, 10). Contemporary updates from the International Union of Immunological Societies (IUIS) place WAS within the expanding catalogue of IEI and emphasize precise molecular diagnosis to inform prognosis and definitive therapy (11, 12).

Early diagnosis remains challenging, as many infants do not exhibit the full triad at onset and may initially be misclassified as immune thrombocytopenia (ITP), especially when thrombocytopenia predominates or when the mean platelet volume (MPV) is within normal limits (13). Indeed, one multi-center study found that roughly 19% of children with WAS were initially managed as having ITP before the correct diagnosis was established (14). Confirmatory diagnosis relies on molecular testing of the *WAS* gene, supported by intracellular WASp flow cytometry, which also aids in identifying female carriers and assessing residual protein expression (15, 16). However, these specialized investigations are often unavailable in developing countries, contributing to prolonged diagnostic latency. Curative treatment by hematopoietic stem-cell transplantation (HSCT) now achieves >90% long-term survival in experienced centers, particularly when performed early and with optimal donor matching (17). Moreover, lentiviral gene therapy has shown encouraging outcomes, restoring WASp expression and immune function with durable clinical benefit and manageable safety profiles (6, 18). Nevertheless, access to such definitive treatments remains uneven globally, particularly in low- and middle-income countries (16).

In North Africa, molecularly confirmed data on WAS are almost absent (19, 20). The Moroccan national registry for inborn errors of immunity indicates that only 29% of patients receive a confirmed genetic diagnosis, reflecting persistent gaps in molecular capacity and healthcare infrastructure (21). Addressing this diagnostic gap is essential for improving disease recognition, facilitating genetic counseling, and expanding access to curative therapy. While WAS patients of Moroccan origin have appeared within broader IEI registry studies (21, 22), no dedicated genetically confirmed WAS cohort from Morocco has been reported to date. We therefore report the first genetically confirmed Moroccan WAS cohort, describing its clinical, immunological, and molecular characteristics.

## Materials and methods

### Study design, patient selection and data collection

This was a mixed retrospective and prospective observational cohort study including patients diagnosed with Wiskott–Aldrich syndrome (WAS) at the Department of Pediatric Hematology (P3) and the Department of Pediatric Clinical Immunology and Infectious Diseases (P1), Abderrahim El Harouchi Mother-Child Hospital, Ibn Rochd University Hospital Center, a tertiary referral center for pediatric hematological disorders and inborn errors of immunity (IEI), receiving referrals from across Morocco. Diagnosis of WAS was guided by the diagnostic framework proposed by the Pan-American Group for Immunodeficiency (PAGID) and the European Society for Immunodeficiencies (ESID) (23), defining WAS as congenital thrombocytopenia with reduced platelet volume, together with either a hemizygous pathogenic or likely pathogenic variant in the *WAS* gene, absent WAS protein (WASp) expression, or a maternal family history of a WAS-related disorder. For this study, the analytic inclusion criterion was a compatible clinical phenotype together with molecular confirmation of a pathogenic or likely pathogenic hemizygous variant in the *WAS* gene. All patients were followed up at our center, and molecular analyses of the *WAS* gene were performed prospectively. Some patients had been followed in our departments for several years for chronic thrombocytopenia and/or clinically suspected WAS before molecular diagnosis became available, while others were enrolled prospectively after genetic testing was introduced. During the study period, 15 patients were evaluated for suspected WAS. Five died before genetic confirmation could be obtained and were therefore excluded. The remaining 10 patients, all with molecularly confirmed WAS, constitute the present cohort. Clinical disease severity was assessed for each patient using the Zhu/Ochs scoring system (15, 24). The score ranges from 0.5 to 5: a score of 1 denotes thrombocytopenia alone; 2, thrombocytopenia with mild eczema and/or minor infections; 3, the classic triad of thrombocytopenia, eczema, and recurrent infections; 4, classic WAS with severe or life-threatening infections; 5A, any of the above with autoimmune complications; and 5M, any of the above with malignancy. Scores were assigned retrospectively based on the most severe manifestations documented during the follow-up period. Detailed clinical characteristics, including medical history, symptoms, laboratory findings, treatment modalities, and disease progression, were collected through comprehensive examination of medical records and hospital archives, and follow-up information was obtained by interviewing families when necessary.

### Immunological and hematological assessment

Initial diagnostic screening included complete blood counts (CBC) to assess platelet count and Mean Platelet Volume (MPV), a key parameter for microthrombocytopenia. MPV was measured by impedance-based automated cell counter (Sysmex XN-series). The reference range for MPV in our laboratory is 7.5–12.5 fL, consistent with published pediatric reference intervals; reduced MPV was defined as a value below 7.5 fL. All platelet counts and MPV values correspond to measurements obtained at initial diagnostic workup, before any platelet transfusion, before any thrombopoietin receptor agonist, and before HSCT. For patients referred after prior empirical corticosteroid therapy, values were re-assessed after steroid washout where contemporaneous records allowed; where unavailable, values are marked ND (not determined). Anti-platelet antibody testing was not performed, as these assays are not routinely available at our institution. Immunoglobulin quantification (IgA, IgG, IgM, and IgE) was performed, alongside lymphocyte subset assessment. All samples were processed in a single laboratory (Immunology Laboratory, CHU Ibn Rochd, Casablanca). Flow cytometric analysis was performed on fresh peripheral whole blood, collected in EDTA anticoagulant tubes and processed within 24 h of collection, using a BD FACSCanto II flow cytometer (BD Biosciences, San Jose, CA) and FACSDiva software. The standard panel comprised anti-CD3-FITC/anti-CD4-PE/anti-CD8-PerCP/anti-CD19-APC for T and B cells, and anti-CD16-FITC/anti-CD56-PE for NK cells. Lymphocytes were gated on a forward-scatter (FSC) vs. side-scatter (SSC) dot plot to exclude debris, monocytes, and granulocytes; subsets were then identified within this gate by their respective surface marker fluorescence. Both relative percentages and absolute counts (cells/mm^3^) were determined for each subset; absolute counts were calculated by multiplying the percentage obtained by the total lymphocyte count from the concurrent CBC. All values were interpreted against published pediatric age-adjusted reference ranges (25, 26). Assessment of intracellular WASp protein by flow cytometry was not performed in this study.

### Genetic analysis

Genomic DNA was extracted from peripheral blood leukocytes using standard phenol-chloroform protocols. The twelve coding exons of the *WAS* gene and their exon-intron boundaries were amplified by PCR using previously published primer pairs and cycling conditions (14, 27). Bidirectional Sanger sequencing was performed on an Applied Biosystems 3500 Genetic Analyzer using BigDye Terminator v3.1 chemistry. Population frequency was assessed using gnomAD v2.1.1; prior variant reports were evaluated via ClinVar (NCBI) and HGMD Professional. For missense variants, in silico pathogenicity was assessed using SIFT, PolyPhen-2, and CADD. For the canonical splice-site variant c.735-2A > T, splicing impact was assessed using SpliceSiteFinder-like and MaxEntScan. Obtained sequences were aligned to the WAS reference sequence NG_007877.1 (GRCh37/hg19) using SeqScape v2.7 (Applied Biosystems), and sequence variants were described according to the Human Genome Variation Society (HGVS) nomenclature. As targeted Sanger sequencing was used rather than next-generation sequencing, sequencing depth (coverage) and bioinformatic variant-calling pipelines were not applicable. Segregation analysis could not be performed because parental DNA samples were unavailable. Methods to detect large intragenic deletions or duplications (MLPA or CNV analysis) were not performed; all ten patients had identifiable point mutations by Sanger sequencing.

### Variant classification

All identified hemizygous variants in the *WAS* gene were classified for pathogenicity using the consensus guidelines established by the American College of Medical Genetics and Genomics and the Association for Molecular Pathology (ACMG/AMP), integrating available evidence including population frequency data (gnomAD v2.1.1), clinical databases (ClinVar, HGMD Professional), published literature, in silico prediction tools for missense variants (SIFT, PolyPhen-2, CADD), and other ACMG evidence criteria (28). Variants were categorized into five tiers: Pathogenic, Likely Pathogenic, Uncertain Significance, Likely Benign, or Benign. Specific criteria were applied with consideration of the X-linked recessive inheritance pattern.

### Ethical approval

This study was conducted in accordance with the ethical principles outlined in the Declaration of Helsinki. The research protocol was reviewed and approved by the Institutional Ethics Committee of Abderrahim El Harouchi Mother-Child Hospital, Ibn Rochd University Hospital Center, Casablanca, Morocco (Approval No. 09/2022). Written informed consent was obtained from the parents or legal guardians of all participating patients for clinical and genetic testing, and for the publication of non-identifiable data in compliance with ethical guidelines.

## Results

### Cohort demographics and clinical manifestations

The cohort comprised 10 male patients with genetically confirmed Wiskott-Aldrich syndrome (WAS) diagnosed between 2017 and 2024. The median age at presentation was 3.3 years (range, 6 months–14 years). Parental consanguinity was documented in 2/10 patients (20%; Cases 4 and 9), and three patients (30%), including twin brothers (Cases 2 and 3), shared a family history of severe infections and hemorrhagic events among maternally related male relatives. Persistent thrombocytopenia was present in all patients (100%). Bleeding manifestations were documented in nine patients (90%), ranging from petechiae and epistaxis to gastrointestinal, ocular, articular, and subconjunctival hemorrhage, oral hemorrhagic bullae, rectal bleeding, and otorrhagia. Infectious manifestations were documented in all 10 patients. Recurrent infections occurred in 9/10 (90%), predominantly respiratory and otologic, whereas Case 5 presented with a single episode of bacterial meningitis at 10 months. Recurrent urinary tract and skin infections, oral candidiasis, and suspected pulmonary mycobacterial infection were also recorded; Case 7 developed bronchiectasis in the context of longstanding recurrent pulmonary infections. Eczema or atopic dermatitis was present in 9/10 patients (90%), and other manifestations included chronic diarrhea in two patients, allergic rhinitis in one, and marked growth retardation in one (Table 1).

**Table 1:** Clinical characteristics, management, outcomes, and HSCT status of Moroccan patients with genetically confirmed wiskott-aldrich syndrome.

Case	Age at Presentation	WAS Score^e^	Medical History	Clinical Presentation	Management	Outcome, Follow-up	HSCT status
1	6 years	5A	ITP^a^ since age 12 months, family history of severe infections and hemorrhage, SLE^b^ diagnosed at age 9 yr	Petechiae, eczema, recurrent infections, positive ANA, proteinuria, hematuria	IVIG, oral prednisolone + hydroxychloroquine (SLE)	Persistent thrombocytopenia; SLE under treatment; Currently 11 years old; 5 years of follow-up.	Not performed (no HLA-matched donor)
2 (Twin 1)	6 months	3	Petechiae since birth; family history of maternal uncle with hemorrhage and severe infection	Petechiae, eczema on face, recurrent infections	IVIG, platelet transfusions, prophylactic TMP-SMX	No major complications. Currently 6 years old; 5 years 6 months of follow-up.	Pending evaluation
3 (Twin 2)	6 months	3	Similar to twin 1; recurrent respiratory infections	Petechiae, eczema, recurrent infections	IVIG, platelet transfusions, prophylactic TMP-SMX	No major complications. Currently 6 years old; 5 years 6 months of follow-up.	Pending evaluation
4	14 years	4	Chronic thrombocytopenia since 5 months; recurrent GI, ocular, articular hemorrhages; consanguineous parents	Petechiae, severe eczema, recurrent otitis, respiratory infections	IVIG, oral prednisolone (severe eczema and refractory thrombocytopenia)	Persistent symptoms. Currently 20 years old; 6 years of follow-up.	Not performed (no HLA-matched donor)
5	1 year	5A	Recurrent epistaxis since 4 months, bacterial meningitis at 10 months, AIHA at age 3 yr^c^	Diffuse petechiae, atopic dermatitis, subconjunctival hemorrhage, splenomegaly, lymphadenopathy	IVIG; rituximab (AIHA)	Favorable post-transplant outcome; sustained remission. Currently 20 years old; 16 years of follow-up post-HSCT.	Performed, full remission
6	1 year 9 months	4	ITP^a^ (steroid-refractory), recurrent UTIs	Petechiae, eczema (atopic dermatitis), oral hemorrhagic bullae, lymphadenopathy, otorrhagia	IVIG, oral corticosteroids (presumed ITP^a^); therapeutic antimicrobials	Recurrent respiratory infections. Currently 5 years old; 3 years 3 months of follow-up.	Pending evaluation
7	13 years	4	ITP^a^ (steroid-refractory), Petechiae since infancy, recurrent pulmonary infections, suspected pulmonary mycobacterial infection^d^	Diffuse eczema, petechiae, chronic cough, bronchiectasis on CT	IVIG, RBC transfusion, oral corticosteroids (presumed ITP^a^)	Deceased at age 37 (severe respiratory infection). 24 years of follow-up post-diagnosis.	Not performed (no HLA-matched donor)
8	5 years 3 months	3	Chronic ITP^a^, recurrent skin infections	Eczematous and impetiginized skin lesions, otitis media, growth retardation (−3 SD), neutropenia§	IVIG, prophylactic TMP-SMX, oral corticosteroids (presumed ITP^a^)	Improved under treatment. Currently 11 years old; 5 years 9 months of follow-up.	Pending evaluation
9	4 years 1 month	3	Recurrent infections, frequent epistaxis, allergic rhinitis and chronic diarrhea, consanguineous parents	Petechiae, epistaxis and rectal bleeding.	IVIG	Stable clinical evolution. Currently 10 years old; 5 years 11 months of follow-up.	Pending evaluation
10	2 years 7 months	3	Recurrent respiratory infections, eczema, chronic diarrhea, oral thrush	Hemorrhagic syndrome including epistaxis and diffuse petechiae.	IVIG, platelet transfusion, oral corticosteroids (eczema); therapeutic antimicrobials	Currently 8 years old; 5 years 5 months of follow-up.	Not performed (no HLA-matched donor)

AIHA, autoimmune hemolytic anemia; ANA, antinuclear antibodies; CT, computed tomography; F/U, follow-up; GI, gastrointestinal; HSCT, hematopoietic stem cell transplantation; IVIG, intravenous immunoglobulin; ITP, immune thrombocytopenic purpura; RBC, red blood cell; SLE, systemic lupus erythematosus; TMP-SMX, trimethoprim-sulfamethoxazole; UTIs, urinary tract infections; yr, year(s).

a ITP denotes the initial clinical working diagnosis assigned before WAS was confirmed. Anti-platelet antibody testing was not performed. In Cases 1, 6, 7 and 8 oral corticosteroids were prescribed as initial management of presumed ITP prior to WAS diagnosis; absence of sustained response to IVIG and corticosteroids prompted genetic testing confirming WAS.

b Diagnostic criteria for SLE (Case 1): ACR/EULAR 2019 criteria met (thrombocytopenia, ANA+, anti-dsDNA+, proteinuria, hematuria, low C3/C4); renal involvement consistent with lupus nephritis.

c AIHA (Case 5): Hb 30–40 g/L, lymphadenopathy, splenomegaly, prolonged fever.

d Suspected pulmonary mycobacterial infection (Case 7): clinical-radiological diagnosis only.

e WAS clinical score (Zhu/Ochs system; range 0.5–5): 1, thrombocytopenia only; 2,   + mild eczema/infections; 3, classic triad; 4, classic WAS with severe complications; 5A, autoimmune complication; 5M, malignancy.

Autoimmune complications occurred in 2/10 patients (20%): Case 1 developed systemic lupus erythematosus (SLE), meeting ACR/EULAR 2019 classification criteria (thrombocytopenia, positive ANA, positive anti-dsDNA, proteinuria, hematuria, low C3/C4), with renal involvement consistent with lupus nephritis, and Case 5 developed confirmed autoimmune hemolytic anemia (AIHA), with hemoglobin levels of 30–40 g/L, splenomegaly, lymphadenopathy, prolonged fever, and a positive direct antiglobulin (Coombs) test. No malignancies were documented during follow-up. All patients had a Zhu/Ochs clinical score of at least 3, consistent with the classic WAS phenotype. Four patients (Cases 1, 6, 7, and 8) had initially been managed for presumed immune thrombocytopenia (ITP) (Zhu/Ochs score of 1 at the time of the initial ITP diagnosis) before molecular confirmation of WAS. All patients received BCG vaccination at birth, and none developed localized or disseminated BCG infection. Detailed patient-level characteristics, management, and outcomes are presented in Table 1, with individual case descriptions in Supplementary File S1.

### Immunological and hematological profiles

All patients presented with persistent thrombocytopenia. Mean platelet volume was available for nine patients (median 8.0 fL, range 6.0–10.0 fL): reduced (below 7.5 fL) in 2/9 patients (22.2%), within the reference range in the remaining 7/9 (77.8%), and not obtained for Case 9. Anemia, microcytic or normocytic, was recorded in nine patients (90%). Frank neutropenia was observed in only one patient (10%, Case 8).

Age-adjusted lymphocyte subset counts varied across the cohort. Absolute counts were reduced for CD3+ T cells in 2/9 patients (22.2%, Cases 6 and 7), CD4+ T cells in 3/9 (33.3%, Cases 4, 6, and 7), CD8+ T cells in 4/10 (40%, Cases 6, 7, 8, and 10), and CD19+ B cells in 2/9 (22.2%, Cases 6 and 9); values not otherwise specified were normal or elevated. CD16+/CD56+ NK-cell counts were normal or elevated in all seven patients tested. Immunoglobulin values were similarly heterogeneous across isotypes. IgE was elevated in 8/9 tested patients (88.9%), including a markedly elevated concentration of 10,221 IU/mL in Case 10, and normal in Case 8. IgA followed the same trend, elevated in 8/10 patients, normal in one, and reduced in one. IgM was elevated in one patient (Case 10), normal in seven, and reduced in two (Cases 4 and 7) (Table 2). IgG in 8 of 10 patients (Cases 1, 2, 3, 4, 5, 7, 9, and 10) was measured after at least one IVIG infusion and reflects exogenous supplementation rather than endogenous production. Pre-IVIG baseline IgG was available only for Cases 6 and 8, elevated in Case 6 and within the age-adjusted reference range in Case 8 (Table 2). Functional immunological assays, including lymphocyte proliferation, intracellular WASp expression, and specific antibody responses, were not performed, as these were not routinely available at our institution during the study period.

**Table 2:** Hematologic and immunologic findings in Moroccan patients with genetically confirmed wiskott-aldrich syndrome.

Patient	Age at assessment	Platelets (×10⁹/L)	MPV (fL)	CD3⁺ (cells/mm^3^)	CD4⁺ (cells/mm^3^)	CD8⁺ (cells/mm^3^)	CD19⁺ (cells/mm^3^)	CD16+/CD56⁺ (cells/mm^3^)	IgE (IU/mL)	IgA (g/L)	IgG (g/L)	IgM (g/L)
1	6 years	10 ↓	8	4400 ↑ (1200–2600)	2890 ↑ (650–1500)	1510 ↑ (370–1100)	ND	ND	ND	1.3 N (0.46–1.50)	12.5* ↑ (5.5–10.2)	1.15 N (0.54–1.53)
2 (Twin 1)	6 months	13 ↓	9	4439 N (1900–5900)	1819 N (1400–4300)	2110 ↑ (500–1700)	1892 N (610–2600)	946 N (160–950)	36 ↑ (<30)	1.41 ↑ (0.27–0.86)	11.36* ↑ (2.4–4.4)	1.10 N (0.34–1.14)
3 (Twin 2)	6 months	24 ↓	8	3636 N (1900–5900)	1515 N (1400–4300)	1394 N (500–1700)	1151 N (610–2600)	396 N (160–950)	38.78 ↑ (<30)	1.41 ↑ (0.27–0.86)	6.97* ↑ (2.4–4.4)	1.08 N (0.34–1.14)
4	14 years	13 ↓	7.6	1132 N (1000–2200)	482 ↓ (530–1,300)	584 N (330–920)	233 N (110–570)	265 N (70–480)	102 ↑ (<30)	7.42 ↑ (0.56–2.03)	12.93* ↑ (6.6–12.2)	0.17 ↓ (0.57–1.62)
5	1 year	26 ↓	7.6	6,074 N (2,100–6,200)	1,341 N (1,300–3,400)	4,496 ↑ (620–2,000)	1,025 N (720–2,600)	394 N (180–920)	102 ↑ (<30)	1.71 ↑ (0.33–1.22)	14.34* ↑ (3.4–6.2)	0.83 N (0.48–1.43)
6	1 year 9 months	8 ↓	10	1,389 ↓ (2,100–6,200)	863 ↓ (1,300–3,400)	471 ↓ (620–2,000)	81 ↓ (720–2,600)	308 N (180–920)	3,792 ↑ (<30)	2.4 ↑ (0.33–1.22)	8.19 ↑ (3.4–6.2)	0.62 N (0.48–1.43)
7	13 years	57 ↓	6.2 ↓	213 ↓ (1,000–2,200)	179 ↓ (530–1,300)	59 ↓ (330–920)	350 N (110–570)	ND	109 ↑ (<30)	8.8 ↑ (0.56–2.03)	16.2* ↑ (6.6–12.2)	0.34 ↓ (0.57–1.62)
8	5 years 3 months	63 ↓	6 ↓	ND	ND	189 ↓ (490–1,300)	1,400 N (390–1,400)	ND	<30 N	0.08 ↓ (0.46–1.50)	7.24 N (5.5–10.2)	1.42 N (0.54–1.53)
9	4 years 1 month	23.3 ↓	ND	4,183 ↑ (1,400–3,700)	1,313 N (700–2,200)	2,842 ↑ (490–1,300)	169.6 ↓ (390–1,400)	523.2 N (130–720)	2,331 ↑ (<30)	4.56 ↑ (0.41–1.41)	13.89* ↑ (4.8–9.0)	1.53 N (0.54–1.53)
10	2 years 7 months	12 ↓	10	2,460 N (1,400–3,700)	2,210 ↑ (700–2,200)	199 ↓ (490–1,300)	498 N (390–1,400)	1,508 ↑ (130–720)	10,221 ↑ (<30)	1.31 ↑ (0.33–1.22)	22.86* ↑ (3.4–6.2)	3.77 ↑ (0.48–1.43)

Platelets: platelet count (×10⁹/L). MPV, Mean Platelet Volume (fL); reference range 7.5–12.5 fL (Sysmex XN-series); values below 7.5 fL defined as reduced. All platelet and MPV values obtained at initial diagnostic workup, before platelet transfusion, before thrombopoietin receptor agonist, and before HSCT. CD3⁺, CD4⁺, CD8⁺, CD19⁺, CD16⁺/CD56⁺ NK cells: lymphocyte counts (cells/mm^3^); absolute counts derived from concurrent CBC. Values interpreted against published pediatric age-adjusted reference ranges (25, 26). IgE: IU/mL. IgA, IgG, IgM: g/L. Values in parentheses represent age-adjusted reference ranges. ↑: above reference range. ↓: below reference range. N: within normal range. ND: not determined. * IgG values marked with an asterisk (*) were measured after intravenous immunoglobulin (IVIG) infusion and therefore reflect exogenous supplementation; they cannot be interpreted as endogenous IgG production. Pre-IVIG baseline IgG was available only for Cases 6 and 8. Flow cytometry performed on a BD FACSCanto II flow cytometer (BD Biosciences) at a single laboratory (Immunology Laboratory, CHU Ibn Rochd, Casablanca). Panel: anti-CD3-FITC/anti-CD4-PE/anti-CD8-PerCP/anti-CD19-APC; anti-CD16-FITC/anti-CD56-PE for NK cells.

### Genetic findings and variant classification

Genetic analysis confirmed a hemizygous pathogenic variant in the *WAS* gene in all ten patients (Table 3), identifying seven distinct variants within the cohort. Truncating or splice-site variants were identified in 8 of 10 patients (80%): four nonsense (p.Gly322*, Cases 1–3; p.Arg321*, Case 7), two frameshift (p.Pro330Leufs*115, p.Arg431Serfs*64), and one canonical splice-site variant (c.735-2A > T, Cases 4–5). The remaining two patients (20%) carried missense variants, c.149T > A (p.Val50Asp, Case 6) and c.383T > G (p.Phe128Cys, Case 8). Six of the seven variants were absent from gnomAD v2.1.1, ClinVar, and HGMD Professional, and are reported here as novel; the variant p.Arg321* (c.961C > T) has prior reports in the literature and in ClinVar (Variation ID 449515; RCV000818878) (24, 29, 30). For the two missense variants, in silico predictions were concordantly pathogenic: p.Val50Asp (SIFT Damaging, PolyPhen-2 Probably Damaging, CADD 34.2) and p.Phe128Cys (SIFT Damaging, PolyPhen-2 Probably Damaging, CADD 35.8), both well above the CADD threshold associated with deleterious effect. The splice-site variant c.735-2A > T was predicted to cause splice-site loss by SpliceSiteFinder-like and MaxEntScan. Segregation analysis could not be performed, as parental DNA samples were unavailable for any family. All identified variants were classified as Pathogenic or Likely Pathogenic according to ACMG/AMP criteria. The genotype–phenotype correlation demonstrated that all variants, including both missense substitutions, resulted in the full-blown classic WAS phenotype, with no patient presenting with the isolated XLT mild phenotype. The full spectrum of mutations, variant types, and pathogenicity classifications, including all evidence codes and in silico scores, is provided in Table 3.

**Table 3:** Genetic mutations, variant types, and ACMG classification in Moroccan patients with wiskott-aldrich syndrome.

Patient	Exon	cDNA#	Protein	Variant type	Domain	ACMG	Prior report
1	10	c.964G > T	p.Gly322*	Nonsense (truncating)	PRR	Pathogenic (PVS1, PM2)	NR
2	10	c.964G > T	p.Gly322*	Nonsense (truncating)	PRR	Pathogenic (PVS1, PM2)	NR
3	10	c.964G > T	p.Gly322*	Nonsense (truncating)	PRR	Pathogenic (PVS1, PM2)	NR
4	Intron 8/exon 9	c.735-2A > T	—	Canonical splice acceptor	PRR	Pathogenic (PVS1)	NR
5	Intron 8/exon 9	c.735-2A > T	—	Canonical splice acceptor	PRR	Pathogenic (PVS1)	NR
6	2	c.149T > A	p.Val50Asp †	Missense	WH1	Likely Pathogenic (PM1, PM2, PP3)	NR
7	10	c.961C > T	p.Arg321*	Nonsense (truncating)	PRR	Pathogenic (PVS1, PM2)	Reported ‡ (24, 29, 30)
8	4	c.383T > G	p.Phe128Cys †	Missense	WH1	Likely Pathogenic (PM1, PM2, PP3)	NR
9	10	c.984_985delinsA	p.Pro330Leufs*115	Frameshift (truncating)	PRR	Pathogenic (PVS1, PM2)	NR
10	11	c.1289dup	p.Arg431Serfs*64	Frameshift (truncating)	VCA	Pathogenic (PVS1, PM2)	NR

# HGVS nomenclature. Reference sequences: NM_000377.3 (cDNA); NP_000368.1 (protein). WH1: WASP Homology 1/EVH1 domain (exons 1–4); WIP binding and protein stability. PRR, proline-rich region (exons 8–10); SH3-binding signaling platform. VCA, verprolin/cofilin/acidic domain (exons 11–12); actin polymerization via Arp2/3. PVS1: null variant in established LOF disease gene. PM1: variant in critical functional domain. PM2: absent from population databases. PP3: concordant computational pathogenicity evidence. NR: not previously reported. All variants identified in this cohort were absent from gnomAD v2.1.1. Six of the seven variants (p.Gly322*, c.735-2A > T, p.Val50Asp, p.Pro330Leufs*115, p.Phe128Cys, and p.Arg431Serfs*64) are not reported in ClinVar or HGMD Professional and are novel. ‡The variant p.Arg321* (Patient 7) has been previously reported in ClinVar (Variation ID: 449515; RCV000818878) and in the published literature [references (24, 29, 30)]. † In silico pathogenicity predictions for missense variants: p.Val50Asp (Patient 6) — SIFT: Damaging (score 0.001), PolyPhen-2: Probably Damaging (score 0.998), CADD: 34.2; p.Phe128Cys (Patient 8) — SIFT: Damaging (score 0.000), PolyPhen-2: Probably Damaging (score 0.999), CADD: 35.8. In silico tools were not applied to truncating or frameshift variants (PVS1 criterion directly applicable). Splicing impact of c.735-2A > T (Patients 4 and 5) was assessed using SpliceSiteFinder-like (pathogenic splice loss) and MaxEntScan (severe reduction of splice site score). Segregation analysis could not be performed because parental DNA samples were unavailable.

### Management and outcomes

Management across the cohort was broadly supportive, with intravenous immunoglobulin used in all ten patients and antimicrobial prophylaxis, transfusion support, and corticosteroids added selectively according to clinical presentation. Corticosteroids were prescribed either empirically for presumed ITP before WAS was diagnosed, or, once confirmed, for specific indications such as autoimmune disease (Case 1, alongside hydroxychloroquine for SLE) or severe eczema (Cases 4 and 10), while rituximab was used in Case 5 for AIHA. Curative therapy remained constrained by donor availability and access to transplant infrastructure. Only Case 5 underwent allogeneic HSCT, performed abroad at age 4 years from an HLA-matched sibling donor; conditioning details were unavailable because the procedure was performed outside our institution. He remains alive and in sustained remission, now aged 20 years with 16 years of follow-up since transplantation. Among the remaining nine patients, four lacked a suitable HLA-matched donor and five were undergoing evaluation for definitive therapy. At last follow-up, all patients were alive except Case 7, who died at age 37 from a severe respiratory infection after 24 years of follow-up without HSCT. Individual treatment, outcome, current age and follow-up duration are detailed in Table 1.

## Discussion

In this study, we present the first genetically confirmed cohort of Wiskott–Aldrich syndrome (WAS) patients from Morocco, addressing a regional gap in the mutational spectrum and clinical management of WAS in North Africa, where genetic diagnostic capacity is still emerging. Our findings indicate that WAS is present but likely under-recognized in Morocco, and that both clinical suspicion and diagnostic infrastructure need strengthening. The variable presentation of the disease frequently delays diagnosis: the classic triad of thrombocytopenia, eczema, and immunodeficiency is often incomplete in infancy, so many patients are first labeled as immune thrombocytopenia (ITP), with WAS eventually confirmed in an estimated 7% to 14% of such cases (27, 31, 32). Hemorrhagic manifestations (petechiae, bruising, mucosal bleeding) are usually the earliest signs, sometimes from the neonatal period, whereas recurrent infections and eczematous dermatitis develop later (14, 32). Our series followed this pattern: nearly all patients bled in early childhood, and most subsequently developed recurrent infections (9/10, 90%) and eczema (9/10, 90%), frequencies comparable to those reported in larger cohorts (14). WAS should therefore be considered in any male infant with unexplained thrombocytopenia and recurrent infections or eczema, particularly when the family history is suggestive, as reported in 30% to 69% of cases (14, 27, 33). In our cohort, three patients (30%) belonged to one extended family in which several maternal uncles had died in infancy from infection or bleeding, a history that raised clinical suspicion of WAS.

The hallmark laboratory abnormality in WAS is microthrombocytopenia, classically defined by a persistently low platelet count with a markedly reduced mean platelet volume (MPV), and small platelet size is described as a nearly universal feature of the disease (34, 35). It is noteworthy, however, that our series challenges this paradigm, only 2/9 (22.2%) of tested patients had a subnormal MPV (below 7.5 fL), whereas the remaining 7/9 patients (77.8%) had MPV within the reference range. This observation is not unprecedented: a recent study documented normal platelet size in 34.4% of 38 genetically confirmed WAS patients (36) and isolated case reports have described the same finding (13, 32, 35). Possible explanations include impedance-based measurement variability in thrombocytopenic samples, age of assessment, partial WAS protein (WASp) expression influencing thrombopoiesis, and retrospective single-time-point sampling. No consistent genotype–MPV correlation has been established in WAS, since normal platelet size has been reported across missense, truncating, and splice-site variants alike (13, 32, 35, 36). Our experience reinforces that a normal MPV does not exclude WAS (36), and clinicians should pursue further evaluation rather than dismiss the diagnosis on platelet indices alone. A comparable deviation from expected teaching was observed for lymphocyte immunophenotyping: elevated absolute CD3+, CD4+, or CD8 + counts were present in several younger patients (Cases 2, 5, 9, and 10, ages 0.5–4 years), whereas WAS is classically associated with progressive T-cell lymphopenia. This pattern is consistent with the known natural history of the disease, in which T-cell lymphopenia typically becomes more pronounced with age rather than being fully established from infancy (37); reactive lymphocytosis during intercurrent infections, common in young children generally, may also have contributed. Flow cytometric analysis of intracellular WASp expression is a useful complementary tool: classic WAS patients typically show absent or extremely low WASp in lymphocytes and platelets, whereas milder X-linked thrombocytopenia (XLT) cases may retain residual WASp expression (37). This assay was not available in our center, and in its absence genetic testing remained the definitive diagnostic modality.

All patients in our series were confirmed by identification of hemizygous pathogenic variants in the *WAS* gene. Consistent with the literature, the mutations were heterogeneous and spanned multiple exons, with a predominance of loss-of-function variants. Truncating mutations (nonsense, frameshift, or splice-site changes) accounted for 80% of our cases, a finding in line with the known mutational spectrum of WAS (30, 38). Consistent with established genotype-phenotype correlations, such null mutations typically abolish WASp production and are associated with the severe “classic” WAS phenotype (30, 38, 39). Given the small cohort size (*n* = 10), this observation should be interpreted as directionally consistent with known patterns rather than as an independent conclusion. Notably, even the two missense variants (p.Val50Asp, p.Phe128Cys, both in the WH1 domain) in our cohort resulted in the full-blown WAS phenotype, illustrating that genotype–phenotype correlation is not absolute. Indeed, certain missense changes can severely disrupt WASp function (e.g., by destabilizing critical domains) and thus present clinically indistinguishable from null mutations (30). No patient presented with the isolated XLT phenotype. Overall, our findings underscore that molecular analysis is indispensable for a conclusive diagnosis, not only to confirm WAS but also to classify the variant, guide family counseling, and potentially inform prognosis.

One of the most challenging aspects of WAS is its propensity for autoimmune complications (3). Published cohorts indicate that roughly 26%–72% of WAS patients experience at least one autoimmune disease during their lifetime (3, 40). In our series, 2 of 10 children (20%) had significant autoimmune phenomena: Case 1 fulfilled criteria for an autoimmune systemic lupus erythematosus (SLE)-like illness, with renal involvement consistent with lupus nephritis, and Case 5 developed confirmed autoimmune hemolytic anemia (AIHA). Lupus is an exceedingly rare association with WAS, only isolated case reports describe SLE in WAS patients (41), making our observation noteworthy. Both of our patients’ autoimmune complications presented early (before age 10), which is consistent with reports that autoimmunity in WAS often manifests in childhood, sometimes even preceding recurrent infections (3). Importantly, the emergence of autoimmunity in WAS is a grave prognostic marker. It has been identified as an independent predictor of worse outcomes and is associated with a higher risk of later malignancies (3, 42). Elevated serum IgM levels have been proposed as a risk factor specifically for AIHA in WAS, although this association is not universal across cohorts (43). The high prevalence of anemia in our cohort (9/10 patients, 90%) warrants comment. A review of clinical and laboratory data suggests that chronic blood loss secondary to recurrent hemorrhagic episodes was the predominant contributing mechanism in most patients, consistent with the microcytic or normocytic pattern observed on CBC. In patients with recurrent infections and prolonged inflammatory disease courses, anemia of chronic inflammation may have contributed additionally. The patient (Case 5) with AIHA, with hemoglobin levels of 30–40 g/L, splenomegaly, lymphadenopathy, and prolonged fever, requiring rituximab, represented the most severe anemic episode in the cohort. AIHA is a well-recognized autoimmune complication of WAS, reported in 8%–36% of patients across published cohorts (3, 38). Systematic iron studies and reticulocyte counts were not obtained across all patients, and the direct antiglobulin test (Coombs) was performed only as part of the diagnostic workup for Case 5, precluding definitive etiological classification in the remaining cases.

WAS patients also face a dramatically increased risk of hematologic malignancies, mainly lymphomas. The cumulative incidence of lymphoma in WAS is approximately 13%–22%, with one large analysis reporting about 15% of patients developing lymphoma by the age of 30 (39). These are frequently EBV-associated B-cell non-Hodgkin lymphomas, though Hodgkin lymphoma and even T-cell lymphomas have been documented (39). The link between autoimmunity and malignancy is especially concerning, chronic immune activation and dysregulation in WAS are thought to drive lymphoproliferation, and indeed WAS patients with a history of autoimmune disease have a significantly higher risk of lymphoma than those without (39, 42). In our cohort, no malignancies were observed, which is unsurprising given the relatively young ages of our patients and the fact that none had prolonged EBV infections. Nonetheless, this zero-incidence should be interpreted with caution due to the small sample size. Vigilant long-term follow-up is warranted, as the malignancy risk in WAS climbs with age.

WAS has historically carried a poor prognosis when managed with conservative therapy. Earlier studies reported a median life expectancy of only around 20 years for untreated patients (44). The major causes of death are severe infections, hemorrhages (often intracranial or gastrointestinal), and complications of malignancies (39, 44). Although survival has improved in recent years due to better supportive measures such as prophylactic antimicrobials, immunoglobulin replacement therapy, aggressive treatment of eczema and infections, and, in selected cases, splenectomy or thrombopoietic agents, these approaches remain only palliative. Allogeneic hematopoietic stem cell transplantation (HSCT) has long been the established curative option for WAS, with long-term survival now exceeding 85%–90% in experienced centers (45). Outcomes are best when HSCT is performed before the age of five years and with a fully HLA-matched sibling donor, while matched unrelated donors now achieve comparable results thanks to improved conditioning and graft management protocols (45, 46). Since December 2025, Waskyra (etuvetidigene autotemcel) has been FDA approved, and it is now authorized in the European Union, for patients aged 6 months and older with a *WAS*-gene mutation for whom HSCT is appropriate and no suitable HLA-matched related donor is available (47). With gene therapy now approved, all classic WAS patients are candidates for definitive therapy (6, 18, 47). In our setting, however, access to definitive therapy remains limited by donor availability, infrastructure, and referral pathways. Indeed, only one of our patients could undergo HSCT, performed abroad due to the absence of local transplant facilities, and he achieved sustained clinical remission with resolution of cytopenia and infections. Meanwhile, supportive care remains the mainstay for patients awaiting curative therapy. All patients in our study received combinations of prophylactic antimicrobials, regular intravenous immunoglobulin (IVIG), platelet transfusions for bleeding, and immunosuppressants for autoimmune or dermatologic complications. This experience underscores the urgent need to improve access to advanced diagnostics and curative modalities in developing countries.

Several limitations of this study warrant acknowledgment. First, the cohort is small (*n* = 10), which limits the statistical power of genotype-phenotype correlation analyses, and all associations reported here should be interpreted with appropriate caution. Second, this was a single-center study conducted at the national tertiary referral center for pediatric hematology and immunodeficiency in Morocco, introducing potential referral and survivorship bias, as our cohort likely enriches for more severe or diagnostically complex presentations. Third, data collection combined retrospective and prospective approaches, and retrospective review resulted in incomplete records for some clinical variables. Fourth, segregation analysis could not be performed because parental DNA samples were unavailable, limiting variant interpretation to evidence available from the proband alone. Fifth, advanced immunological testing, including intracellular WASp flow cytometry, lymphocyte proliferation assays, and specific antibody responses, was not available at our center; absolute lymphocyte counts alone do not fully capture the immune synapse defects characteristic of WASp-deficient T cells, limiting functional characterization. Sixth, MLPA/CNV analysis for large intragenic rearrangements was not performed, as all ten patients had identifiable point mutations by Sanger sequencing. Seventh, IgG measurements in 8 of 10 patients were obtained after IVIG infusion and cannot be interpreted as reflecting endogenous immunoglobulin production. Eighth, Coombs testing was not systematically performed across the cohort. Ninth, platelet morphology was not systematically confirmed by peripheral blood smear examination before platelet transfusion, and MPV assessment therefore relied on automated analyzer values. Finally, access to curative therapy remained severely limited by the absence of national pediatric transplant infrastructure, reflecting a systemic gap in access rather than individual clinical ineligibility. Despite these limitations, the overall outcome of our Moroccan series remains cautiously encouraging: most patients are alive and under follow-up, one achieved sustained remission after HSCT, and one patient died of refractory pulmonary infection.

## Conclusion

In conclusion, our study represents the first genetically confirmed Moroccan series of Wiskott-Aldrich syndrome (WAS), highlighting both the diagnostic and therapeutic challenges faced in resource-limited settings. Aside from the unexpectedly high frequency of normal MPV, the cohort broadly resembled previously reported classic WAS series in its burden of early bleeding, recurrent infections, eczema, and severe supportive-care needs. Truncating and splice-site variants predominated, but genotype–phenotype conclusions should remain cautious in a cohort of this size. Despite advances in supportive management, outcomes remain largely dependent on access to curative options. The survival of Case 7 to the age of 37 without HSCT reflects the outcome achievable with intensive supportive management, but he ultimately died of a preventable complication that curative therapy could have averted. In our series, only one patient benefited from hematopoietic stem cell transplantation (HSCT) performed abroad, achieving sustained remission, while others continue on supportive therapy. These data reinforce the need to pursue molecular testing in clinically compatible male patients even when MPV is normal, and to refer all confirmed patients for assessment of definitive therapy, including HSCT and, where available and appropriate, gene therapy.

## Data Availability

The datasets for this article are not publicly available due to concerns regarding participant/patient anonymity. Requests to access the datasets should be directed to the corresponding author.
