# Supplementary material for: Wiskott-Aldrich syndrome: clinical, immunological, and genetic characterization of the first Moroccan cohort
Source: Front Pediatr. 2026 Aug 3;14:1908492. doi: 10.3389/fped.2026.1908492 (PMC13478139; doi:10.3389/fped.2026.1908492)
Supplement: Supplementary file 1 [file Supplementaryfile1.docx]

**Supplementary File S1. Individual Case Descriptions**

*Wiskott-Aldrich Syndrome: Clinical, Immunological, and Genetic Characterization of the First Moroccan Cohort*

El Alaoui El Hanafi M, Benhsaien I, Maani K, El Bakkouri J, Benmoussa AY, Benajiba N, Outabounte A, Errami A, Ailal F, Bousfiha AA, Abkari A.

# Case Descriptions

**Case 1:**

A 6-year-old boy was admitted to our pediatric hematology unit for evaluation of chronic immune thrombocytopenic purpura (ITP) present since 12 months of age. There was no parental consanguinity. The family history revealed twin cousins with similar manifestations and two maternal uncles who died from severe infections and hemorrhage (Table 1). On examination, he had petechiae and intermittent eczema. Blood analysis showed leukocytosis (14 × 10⁹/L), hemoglobin 110 g/L, and platelets 10 × 10⁹/L with a normal mean platelet volume (MPV) of 8 fL (Table 2). Lymphocyte immunophenotyping showed elevated CD3⁺, CD4⁺, and CD8⁺ T-cell counts. Despite IV immunoglobulin and corticosteroids, no improvement was observed. Genetic sequencing identified a hemizygous WAS variant c.964G>T (p.Gly322*) (Table 3). At 9 years of age, he developed prolonged unexplained fever with positive antinuclear and anti-dsDNA antibodies, proteinuria (2 g/day), hematuria, elevated creatinine (239 μmol/L), and low complement levels (C3 and C4). Systemic lupus erythematosus (SLE) was diagnosed based on these findings. The patient remains under medical management.

**Cases 2 and 3 (Twin brothers):**

Twin male infants, six months of age, were admitted for persistent petechiae noted since birth. There was no parental consanguinity, but the family history revealed two maternal uncles who had died from severe infection and intracranial hemorrhage, and a cousin with refractory immune thrombocytopenia (Table 1). On examination, both presented diffuse petechiae and facial eczema. For Twin 1, complete blood count showed WBC 12.2 × 10⁹/L, hemoglobin 99 g/L, and platelets 13 × 10⁹/L with a normal mean platelet volume (MPV) of 9 fL. Twin 2 had WBC 9.7 × 10⁹/L, hemoglobin 77 g/L, and platelets 24 × 10⁹/L with MPV 8 fL (Table 2). Lymphocyte immunophenotyping showed normal total T-cell (CD3⁺) and B-cell (CD19⁺) counts in both twins, with an isolated elevation of CD8⁺ T cells in Twin 1; immunoglobulin levels showed elevated IgE and IgA with normal IgM. Given the combination of eczema, early-onset thrombocytopenia, and recurrent infections, an inherited platelet disorder was suspected. Molecular analysis confirmed a hemizygous pathogenic WAS mutation c.964G>T (p.Gly322*) in both twins (Table 3). They received supportive intravenous immunoglobulin (IVIG) therapy and prophylactic trimethoprim-sulfamethoxazole (TMP-SMX) with partial improvement. At present, both are two years old and remain clinically stable under continued medical follow-up.

**Case 4:**

A 14-year-old boy was referred for evaluation of chronic thrombocytopenia documented since infancy. His medical history included multiple bleeding episodes involving the gastrointestinal tract, eyes, nose, and hip joints, along with recurrent respiratory and ear infections (Table 1). His parents were consanguineous but asymptomatic. Physical examination revealed diffuse petechiae and eczema. Laboratory investigations showed WBC 10.5 × 10⁹/L, hemoglobin 95 g/L, and platelets 13 × 10⁹/L with a mean platelet volume of 7.6 fL (Table 2). Bone marrow studies were non-contributory. Immunophenotyping showed a normal total T-cell (CD3⁺) count with reduced CD4⁺ and normal CD8⁺ subsets. Serum immunoglobulins showed elevated IgG (measured after IVIG) and elevated IgA, with reduced IgM. The patient received corticosteroids and IVIG without hematologic response. Genetic analysis confirmed a hemizygous splice-site mutation c.735-2A>T in the WAS gene (Table 3), consistent with the diagnosis of Wiskott–Aldrich syndrome. He remains under supportive care and clinical follow-up.

**Case 5:**

A 1-year-old boy was hospitalized for diffuse petechiae, subconjunctival hemorrhage, epistaxis and recurrent respiratory infections that began at 4 months of age. His history included repeated respiratory infections and atopic dermatitis, with a documented episode of bacterial meningitis successfully treated at 10 months (Table 1). Physical examination revealed facial eczema and petechiae on the lower limbs. Hematologic testing showed WBC 17.1 × 10⁹/L, hemoglobin 90 g/L, and platelet count 26 × 10⁹/L, with an MPV of 7.6 fL (Table 2). Immunophenotyping demonstrated elevated CD8⁺ lymphocytes with normal CD4⁺, and immunoglobulin analysis revealed increased IgE, IgG, and IgA levels. At 3 years of age, he developed autoimmune hemolytic anemia (hemoglobin 30–40 g/L) with lymphadenopathy, splenomegaly, and prolonged fever. He was treated with red blood cell and platelet transfusions, IVIG, and rituximab, which led to marked clinical improvement. Genetic testing, performed with parental consent, confirmed a hemizygous splice-site mutation c.735-2A>T in the WAS gene (Table 3). At 4 years of age, the patient underwent hematopoietic stem cell transplantation (HSCT) in France from an HLA-matched sibling donor, with a favorable post-transplant outcome and sustained remission.

**Case 6:**

A 21-month-old boy was evaluated for persistent epistaxis, otorrhagia, petechial rash, and fever lasting six days. His medical history included atopic dermatitis from 15 months of age, immune thrombocytopenia requiring platelet transfusions, and recurrent urinary tract infections (Table 1). Clinical examination revealed widespread petechiae, purpura, oral hemorrhagic bullae, cervical lymphadenopathy, fetid otorrhea, and pulmonary crackles. Laboratory findings indicated microcytic hypochromic anemia (hemoglobin 92 g/L), severe thrombocytopenia (8 × 10⁹/L) with normal MPV (10 fL), and leukocytosis. Lymphocyte subpopulation counts were CD3⁺ 1,389/mm³, CD4⁺ 863/mm³, CD8⁺ 471/mm³, CD16⁺/CD56⁺ 308/mm³, and CD19⁺ 81/mm³, while immunoglobulins were markedly elevated (Table 2). Bone marrow aspiration revealed eosinophilia and increased megakaryocytes, and HIV serology was negative. Genetic analysis identified a hemizygous missense variant c.149T>A (p.Val50Asp) in the WAS gene (Table 3). The patient received intravenous immunoglobulin, antibiotics, and corticosteroids, with partial clinical improvement. At 5 years of age, he continues to experience recurrent respiratory infections and eczema and is under evaluation for hematopoietic stem cell transplantation.

**Case 7:**

A 13-year-old boy was referred for hemarthrosis and suspected pulmonary mycobacterial infection (microbiologically unconfirmed). His medical history revealed petechial and purpuric rashes since infancy, recurrent epistaxis, and hemorrhage following circumcision. He also presented with several respiratory infections and a steroid-resistant immune thrombocytopenia (Table 1). On examination, he exhibited diffuse petechiae and extensive, treatment-resistant eczematous lesions (Supplementary Figure 1A), with a productive cough and crackles predominantly at the lung bases. Chest radiography showed alveolar opacity in the right lower lobe and mediastinal lymphadenopathy (Supplementary Figure 1B), while computed tomography revealed bilateral bronchiectasis and right-sided atelectasis (Supplementary Figure 1C). Laboratory findings indicated microcytic normochromic anemia and thrombocytopenia (57 × 10⁹/L) with a low mean platelet volume (6.2 fL). Immunological assessment showed lymphopenia and elevated serum IgG (16.2 g/L, measured after IVIG) and IgA (8.8 g/L) levels (Table 2). A hemizygous c.961C>T (p.Arg321*) nonsense mutation in the WAS gene was confirmed (Table 3). Despite treatment with systemic corticosteroids, red-blood-cell transfusions, and intravenous immunoglobulins, the patient’s condition gradually deteriorated, and he died at the age of 37 years from a severe respiratory infection.

**Supplementary Figure 1. Clinical and radiological findings in Case 7. (A) Chronic eczematous lesions of the lower limb; (B) Chest X-ray showing alveolar opacity of the right lower lobe with mediastinal adenopathy; (C) Chest CT scan revealing bilateral bronchiectasis with right-sided atelectasis.**

**Case 8:**

A 5-year-and-3-month-old boy was admitted for chronic immune thrombocytopenic purpura and recurrent skin infections since three months of age. There was no history of consanguinity, diarrhea, or oral candidiasis, and no similar familial cases were reported. Physical examination revealed erythematous, scaly, crusted, and impetiginized lesions on the face and limbs, with additional pustular eruptions on the forehead and behind the left ear, purpuric and ecchymotic lesions, and left-sided purulent otitis media (Supplementary Figure 2). His growth parameters were below the expected range (−3 SD) (Table 1). Laboratory findings showed microcytic hypochromic anemia (110 g/L), refractory thrombocytopenia (63 × 10⁹/L), and a low mean platelet volume (6 fL). Immunoglobulin testing revealed decreased IgA (0.08 g/L) and normal IgG (7.24 g/L), while lymphocyte subpopulation analysis demonstrated markedly reduced CD8⁺ cells (189/mm³) and normal CD19⁺ B cells (1,400/mm³) (Table 2). A hemizygous missense mutation c.383T>G (p.Phe128Cys) in the WAS gene was identified (Table 3). Treatment with intravenous immunoglobulin and prophylactic trimethoprim–sulfamethoxazole led to clinical improvement, with resolution of fever and reduced infection frequency.

**Supplementary Figure 2. Chronic eczematous and impetiginized skin lesions observed in Case 8.**

**Case 9:**

A 4-year-old boy, the youngest of three siblings born to consanguineous parents, was evaluated for recurrent infections and bleeding episodes. The family history revealed several early deaths among maternal uncles (male infants at 1 year and 7 days) and one stillbirth. Clinically, he presented with recurrent upper respiratory tract infections every 2–3 months, chronic diarrhea, allergic rhinitis, conjunctivitis, easy bruising, epistaxis, petechial purpura, rectal bleeding, and pallor. Physical examination confirmed generalized petechiae and mild hepatosplenomegaly. Laboratory evaluation showed normocytic normochromic anemia (Hb 100 g/L), thrombocytopenia (23.3 × 10⁹/L), leukocytosis with eosinophilia, and elevated immunoglobulins (Table 2). Lymphocyte immunophenotyping revealed increased CD3⁺ and CD8⁺ cells, normal CD4⁺ counts, decreased B lymphocytes, and normal NK cells. Genetic testing confirmed a hemizygous c.984_985delinsA (p.Pro330Leufs*115) mutation in the WAS gene (Table 3), establishing the diagnosis of Wiskott-Aldrich syndrome. The patient was treated with intravenous immunoglobulin, which led to significant clinical improvement.

**Case 10 :**

A 2-year-and-7-month-old boy was admitted for evaluation of persistent bleeding and recurrent infections. Since the age of four months, he had suffered from repeated respiratory infections treated with antibiotics and intermittent corticosteroids. Eczema appeared early in infancy and recurred after stopping topical corticosteroids. He also experienced episodes of oral thrush and chronic diarrhea. At admission, he presented with severe hemorrhagic syndrome characterized by right-sided epistaxis, petechiae, ecchymoses, hemorrhagic bullae on the oral mucosa, and eczematous lesions (Table 1). Hematological investigations showed microcytic hypochromic anemia (Hb 100 g/L) and thrombocytopenia (12 × 10⁹/L) with a normal mean platelet volume (10 fL). Bone marrow examination was unremarkable. Immunoglobulin assays revealed markedly elevated IgE (10,221 IU/mL), while flow cytometry showed elevated CD4⁺ T cells with reduced CD8⁺ T cells and normal CD19⁺ B-cell counts (Table 2). Genetic analysis identified a c.1289dup (p.Arg431Serfs*64) frameshift mutation in the WAS gene (Table 3), confirming the diagnosis of Wiskott-Aldrich syndrome. The patient was treated with intravenous immunoglobulin, platelet transfusions, corticosteroids, and antibiotics, and has not undergone HSCT owing to the absence of a suitable HLA-matched donor.
